# Report of a Case of Puerperal Eclampsia, with Notes

**Published:** 1889-11

**Authors:** B. F. Kingsley

**Affiliations:** San Antonio, Tex.


### REPORT OF A CASE OF PUERPERAL ECLAMPSIA, WITH NOTES.

BY B. F. KINGSLEY, M. D., SAN ANTONIO, TEX.

Read at annual meeting West Texas Medical Society, and referred to this, the official organ, for publication.

I REGRET that my limited time has prevented me from presenting the case in a more acceptable form; but I cannot forego the opportunity to offer a sketch of the case, imperfect and hasty as it is, for a twofold reason: first, for its intrinsic history; and, second, to illustrate and emphasize the value of treatment of this disease by veratrum viridi,—a remedy I believe to be more efficacious than any other, perhaps all others, in a vast majority of cases.

I say majority, for I recognize the probable fact that all cases of puerperal convulsions are not due to one and the same cause; therefore the same treatment would not be applicable. Each case must be studied independently, and the varying conditions of temperament, pulse, respiration, temperature, skin, bowels, bladder and urine noted, and treatment governed accordingly.

Yet in spite of this diversity in symptomatology and etiology, I believe that certain pretty regular, well difined symptoms characterize a large majority of cases, these, of course, differing in character and severity. I am led to this conclusion from the fact that albuminuria is more largely discussed by authors in treating of this disease than any other cause. This is caused by reasons which will appear later on, and in turn produce ultimately the convulsions and other phenomena. And also for the reason that in all the cases I have ever seen, six in number, this was a conspicuous symptom among others, which inevitably follow sooner or later; and I uphold and laud the virtues of veratrum because I have used it in four cases and saved my patients, whereas in the other two, treated with chloral hydrate, and both post partum, where the mortality is least, I lost both.

Was called at 12:30 p. m., September 28, 1888, by my sister, Dr. Josephine Kingsley, to assist in a case of labor complicated with convulsions. After some delay I arrived an hour later, to find the woman, a small, well nourished Mexican, delivered, but in a convulsion. She had been given chloroform during the hour or hour and a half necessary to deliver. I at once gave twenty grains of antipyrine hypodermically that happened to be in the house, and in fifteen minutes ten grains more, and ordered some Norwood's tr. veratrum viridi. In the mean time I obtained the following history: Labor pains and convulsions came on simultaneously at 4 a. m., September 28, 1888, and an irregular physician was called, who, without making any examination, informed the patient's friends she would be confined in about five days, and proceeded to put into practice the masterly plan of expectancy, by giving chloroform through seven weary hours, with the woman at term, and every moment the convulsions increasing in force and frequency. At 11 a. m. a change of physicians was decided upon, and my sister, Dr. Josephine Kingsley, was sent for, who responded to the call promptly. She at once instituted an examination, and found the os only sufficiently dilated to admit the index finger, and rather rigid. The patient had had, they said, nearly one hundred convulsions, which number was doubtless considerably overestimated. My estimate was eighty. At this crisis there seemed to be only one thing to do, and that promptly: dilate and empty the womb as speedily as possible. This she succeeded in accomplishing after the laborious effort of an hour and a half. After dilating sufficiently, the head was found to present in the R. O. A. position, the forceps were applied, and a large, living male child delivered, with a median perineal laceration. The convulsions continued. Shortly after this I arrived, and simultaneously Dr. J. Kingsley was called to attend another labor case, and I took charge of the patient.

After giving her the antipyrine before mentioned, and the veratrum having arrived, I gave five m. hypodermically. In pursuing my inquiries, I found she had suffered in no unusual way during gestation, save from moderate swelling of feet, which did not inconvenience her; that she was a primipara, married sixteen months; miscarried three months after marriage; pulse 144, and bounding; respiration 30; temperature 102; tongue heavily coated; skin dry, patient profoundly comatose. In about fifteen minutes, I gave her a second dose of five m., and a third dose of five m. in fifteen minutes more. By this time the pulse became perceptibly affected, but I gave three m. more, and proceeded to examine the urine, a sample of which I obtained with the catheter. A small quantity placed in a clean bottle and heated over a lamp, solidified completely.

I now returned to my patient, who had had no convulsion after the first dose of veratrum had been given, to find her pulse barely perceptible. I was much alarmed, lest she would die promptly from the effects of my favorite remedy. I hurriedly injected into her arm half a drachm of whiskey, which I repeated three times within half an hour. With this her pulse regained its regularity, and considerable volume. I remained an hour longer, and when I left her pulse was sixty, and not quite so full and strong as normal. I returned two hours later, with Dr. J. Kingsley, and repaired the laceration with three stitches, and sprinkled it with iodoform. Found the pulse nearly normal, and left directions that the veratrum should be given, five drops every three or four hours, should the pulse get more frequent. I left directions for a warm carbolized vaginal injection to be given night and morning.

Upon my return the next morning, I found the pulse 100. She was comatose, but had rested quietly during the night. Continued veratrum, and prescribed acetate of potash, F. E. buchu, and tincture hyoscyamus three times a day. By night of second day there were evidences of returning consciousness. She was pretty restless during the day. The second night I gave her bromides. The third day she was semi-conscious. The same treatment was continued, in diminishing quantities, for about two weeks, during which she continued to improve, and was nearly ready to get up, though not without a trace of mental inco-ordination, when she was siezed with mania, under which she talked incessantly, and was very restless. Her temperature, that had been normal for some time, went up to a hundred and three; her tongue became coated, the perineal rent had healed, and there was no way to account for this attack, unless by charging it to a bilious attack. In a few days, from the effect of a mercurial purge and quinine, she became better. Her fever left, and pulse improved. This mental aberation continued, in a mild form, for two weeks, when she became rational, and in two weeks more was up and out, and to-day is well and hearty.

The child died four months after birth from inanition.

Now, the point I wish to emphasize and uphold is, that certain symptoms or succession of phenomena characterize eclampsia with as much regularity, as any set of symptoms characterize any other given disease; 1st, That owing to undue pressure upon the ureters, or one or both kidneys, or upon the pelvic nerves or vessels, whereby the nervous or vascular supply is deranged, there sooner or later appears albumen in the urine. That albumen is found in the urine in other condition, as in Bright's disease, for instance, without convulsions, I can not deny, nor have I time to discuss, but suffice it to say, that owing to the renal derangement due to pressure, in itself a constant irritant, and of great significance, there finally appears renal hyperæmia, then inflammation and albuminurea and probable absorption of other urinary products into the blood, possibly the formation of ptomaines, nntil finally the convulsion occurs. That the pressure alone on some pelvic tissue is sufficient to cause convulsions regardless of the condition of the kidney, is maintained by Prof. A. F. A. King, of Washington, who claims that postural treatment alone will cure many cases; that the head should be raised out of the pelvis on to one of the illiac fossae where it normally belongs, and there maintained by the knee-elbow position. The postural treatment as a prophylactic cannot be too highly commended; in fact, I feel certain that this alone would prevent the development of many cases.

But given the symptoms, present in this case, what should be done? In this case we have a strong bounding pulse of 144. This is the true guide and indication for the use of veratrum; it indicates great vascular and nerve tension. When this is present in any epileptiform attack, it will be found prompt and effectual. Dr. King was perhaps first to suggest its use theoretically, but has never used it practically. To Dr. Fern, of Brooklyn, belongs the credit of having brought its virtues to notice in 1871; and to have shown no convulsion can occur, if the pulse is kept below 60, and this is an easy matter; and further, its apparently dangerous effects may be promptly and safely combatted with stimulants. Its therapeutic effects are visible in half an hour. It reduces the pulse, relieves the terrible vascular and nervous tension more promptly and perfectly than anything else, upon which many deaths from apoplexy and asphyxia depend. This is accomplished, probably, on the theory of Wood, that a person is bled copiously into his own veins by its action. On this theory it should displace blood letting. Of course, it is not intended that the condition of the patient in other respects should be ignored.

What policy should govern our action in anti-partum cases? We have seen how the expectant plan worked by the aid of chloroform in this case. As convulsions prior to the six and one half month, or in other words, before the viability of the child, rarely occurs, we can, as I believe we should, in most cases empty the womb as soon as possible, and still have the highest regard for mother and child.

I have not had time to touch upon numerous exceptions, nor to discuss different methods of treatment, all of which have their proper places and advocates; but there is one more point I have not made sufficiently plain to suit myself, and that is, that the normal sensibility, or irritability, or that resident faculty, by which an organism becomes conscious, or by virtue of which it takes notice of pain, or of mental or physical impressions, acting through the senses, which enables them to endure pain and resist the influence of wounds of poisons, and the evil and untoward consequences of things injurious, and which differs widely in the individual and in the race. It is this force, or the lack of it, which will account for the ease with which some are seized, without much provocation, and why others withstand the assaults of pressure of albuminurea, of nephritis, etc., with apparent impunity.

